# Baicalin Attenuates Oxygen–Glucose Deprivation/Reoxygenation–Induced Injury by Modulating the BDNF-TrkB/PI3K/Akt and MAPK/Erk1/2 Signaling Axes in Neuron–Astrocyte Cocultures

**DOI:** 10.3389/fphar.2021.599543

**Published:** 2021-06-21

**Authors:** Changxiang Li, Conglu Sui, Wei Wang, Juntang Yan, Nan Deng, Xin Du, Fafeng Cheng, Xiaona Ma, Xueqian Wang, Qingguo Wang

**Affiliations:** ^1^ School of Traditional Chinese Medicine Department, Beijing University of Chinese Medicine, Beijing, China; ^2^ Third Affiliated Hospital, Beijing University of Chinese Medicine, Beijing, China

**Keywords:** ischemic stroke, baicalin, astrocyte, coculture, BDNF/TrkB pathway, apoptosis, inflammation

## Abstract

**Background:** Baicalin (BCL), a candidate drug for ischemic stroke, has been indicated to protect neurons by promoting brain-derived neurotrophic factor (BDNF). However, the cellular source of BDNF release promoted by baicalin and its detailed protective mechanism after ischemia/reperfusion remains to be studied. The aim of this study was to investigate the neuroprotective mechanisms of baicalin against oxygen–glucose deprivation/reoxygenation (OGD/R) in a neuron–astrocyte coculture system and to explore whether the BDNF-TrkB pathway is involved.

**Methods and Results:** A neuron–astrocyte coculture system was established to elucidate the role of astrocytes in neurons under OGD/R conditions. The results demonstrated that astrocytes became reactive astrocytes and released more BDNF in the coculture system to attenuate neuronal apoptosis and injury after OGD/R. BCL maintained the characteristics of reactive astrocytes and obviously increased the expression of cyclic AMP response element-binding protein (CREB) and the levels of BDNF in the coculture system after OGD/R. To further verify whether BDNF binding to its receptor tyrosine kinase receptor B (TrkB) was required for the neuroprotective effect of baicalin, we examined the effect of ANA-12, an antagonist of TrkB, on NA system injury, including oxidative stress, inflammation, and apoptosis induced by OGD/R. The results showed that treatment of NA systems with ANA-12 significantly attenuated the neuroprotection of BCL. The phosphatidylinositol 3-kinase (PI3K)/protein kinase B (Akt) and mitogen-activated protein kinase (MAPK)/extracellular signal-regulated kinase (ERK) pathways are two important downstream cascades of signaling pathways activated by BDNF binding to TrkB. We investigated the expressions of TrkB, PI3K, Akt, MAPK, and ERK. The results demonstrated that baicalin significantly increased the expressions of TrkB, PI3K/AKT, and MAPK/ERK.

**Conclusion:** The neuroprotective effects of baicalin against oxidative stress, inflammation, and apoptosis were improved by astrocytes, mainly mediated by increasing the release of BDNF and its associated receptor TrkB and downstream signaling regulators PI3K/Akt and MAPK/ERK1/2.

## Introduction

Stroke is a predominant cause of permanent disability and death worldwide (Katan and Luft 2018). Ischemic strokes (ISs) account for approximately 87% of all strokes (Dabrowska et al., 2019). Even after decades of research, recombinant tissue-type plasminogen activator (Rt-PA) is the only standard therapeutic drug approved by the FDA to treat ISs. Only a few patients (2–5%) can receive Rt-PA thrombolytic therapy because of the narrow time window (McDermott et al., 2019). Therefore, there is an increasing focus on developing neuroprotective agents.

In a great number of studies, neuroprotective agent development experiments only target neurons that lack contact with other types of brain cells and cannot reflect the characteristics of the *in vivo* brain. Other brain cells have critical specific signals and of execution cascades that can also promote neuronal recovery (Liu and Chopp 2016; Venkat et al., 2018; Sun et al., 2019). Moreover, all cell types in the brain may be destroyed after ISs, and the relative balance among them is broken (Dalkara et al., 2016; Liu and Chopp 2016; Venkat et al., 2018). Interactions between these cells regulate complex brain functions under pathological conditions and further aggravate brain damage (Venkat et al., 2018). Therefore, the development of neuroprotective agents aims to expand their effect from protecting pure neurons to protecting multiple types of cells and coordinating their interactions.

Cocultivation systems are increasingly used for cell–cell interactions and for studying drugs to protect neurons indirectly by targeting one type of cell after ischemia-reperfusion (Xue et al., 2013; Yamamizu et al., 2017; Bhalerao et al., 2020; Heymans et al., 2020). Astrocytes, a critical structural and functional part of the neurovascular unit, play an essential role in maintaining normal brain function and responses to ischemic lesions (Liu and Chopp 2016). After an ischemic stroke, astrocytes contribute to neurogenesis, synaptogenesis, and axonal remodeling, thereby promoting neurological recovery (Liu and Chopp 2016). The pivotal involvement of astrocytes designates them as excellent therapeutic targets to improve functional outcomes following a stroke (Sun et al., 2019). Astrocytes and neurons interact closely under physiological and pathological conditions after an IS (Liu and Chopp 2016). In this study, we established a relevant and convenient neuron–astrocyte coculture system to recapitulate this complex interaction.

Baicalin (BCL) is an effective drug candidate for the treatment of ISs. BCL, isolated from the root of *Scutellaria baicalensis* Georgi, is an important flavonoid compound as a potential neuroprotective agent (Kandhasamy et al., 2018). A number of *in vitro* and *in vivo* studies have demonstrated that BCL possessed various pharmacological mechanisms, including antioxidative stress, anti-excitotoxicity, antiapoptotic, and anti-inflammatory effects, inducing neuroregeneration and promoting the expression of neurotrophic factors (Zhu et al., 2012; Fafeng et al., 2013; Liang et al., 2017; Kandhasamy et al., 2018). Moreover, BCL exhibits a variety of beneficial effects in the central nervous system by protecting neurons, astrocytes, Schwann cells, brain microvascular endothelial cells, and the blood–brain barrier from ischemia-reperfusion injury (Shi et al., 2017; Wenpu et al., 2017; Kandhasamy et al., 2018; Song et al., 2020). In particular, a previous study indicated that treatment with BCL remarkably promoted the expression of BDNF in a global cerebral ischemia/reperfusion injury model (Cao et al., 2011).

BDNF, one of the most important neurotrophic factors, promotes neuronal survival and repairs brain damage following ischemia/reperfusion (Liu et al., 2020). A previous study showed that BDNF exerted neuroprotective effects against ischemic stress and inflammation, and decreases apoptosis. TrkB is an endogenous receptor of BDNF with high affinity. The intracellular domain of the tyrosine residue is autophosphorylated after BDNF binds to TrkB, resulting in ligand-induced dimerization of each receptor, which subsequently activates downstream cascades of signals from three signaling pathways activated by TrkB: the PI3K/Akt, MAPK/ERK, and PLCγ pathways (Liu et al., 2020). However, the precise protective mechanisms of BCL during cerebral ischemia/reperfusion through BDNF-TrkB remain to be studied.

Accumulating studies have suggested that BDNF is produced in neurons, but astrocytes are also an important source of BDNF in the brain. BDNF is released from astrocytes via exocytosis to regulate the function of neighboring neurons (Nobukazu et al., 2015; Hong et al., 2016). The coculture system of astrocytes and neurons is suitable for studying the effect of neuroprotective agents on neurons by promoting the release of BDNF from astrocytes (Quesseveur et al., 2013; Hong et al., 2016). Therefore, we established a coculture system of neurons and astrocytes to investigate the neuroprotective mechanisms of BCL against neuronal injury induced by OGD/R and to explore whether the BDNF-TrkB-PI3K/Akt and BDNF-TrkB-MAPK/ERK signaling pathways were involved.

## Materials and Methods

### Animals

Newborn Sprague–Dawley (SD) rats of indicated days were purchased from Beijing Weitong Lihua Experimental Animal Technology (license no. SCXK 20160006, Beijing, China). Animal welfare and experimental procedures were carried out in accordance with the National Institutes of Health Guide for the Care and Use of Laboratory Animals and were approved by the Ethics Committee of Experimental Animals of Beijing University of Chinese Medicine (BUCM-3-2016040201-2003).

### Isolation and Culture of Primary Neurons and Astrocytes

Primary neurons and astrocytes were obtained as in our previous study (Li et al., 2019a; Li et al., 2019b). Briefly, brains were dissected from 0- to 24-hour-old (neurons) and 2- to 3-day-old (astrocytes) SD rats. The brains were cut into halves, and the meninges were removed with tweezers. The cortices were dissected away, and much of the white matter was removed. The cortex was digested at 37°C for 20 min with 0.125% trypsin-EDTA (Sigma-Aldrich, St. Louis, MO, United States). The digested cortical tissues were terminated and homogenized to single-cell suspensions with Dulbecco’s modified Eagle’s medium (DMEM)/F12 medium (Gibco-BRL, Grand Island, NY, United States) containing 10% FBS (Gibco-BRL). Then, the suspension was filtered through a 70-μm cell strainer and centrifuged at 800 rpm for 5 min. The precipitate of neurons was resuspended with Neurobasal-A medium (Gibco-BRL) containing 10% FBS, 2% B27 (Invitrogen; Thermo Fisher Scientific, Inc.), 0.25% GlutaMAX (Gibco-BRL), and 1% penicillin/streptomycin (P/S, Gibco-BRL). Cells were then seeded at a density of 1 × 10^6^ cells/ml into 0.01% poly-l-lysine (PLL, Sigma-Aldrich) precoated dishes. The medium was changed to non-serum formula after seeding for 6–8 h. The precipitate of astrocytes was resuspended with DMEM/F12 (Gibco-BRL) containing 10% FBS (Gibco-BRL) and 1% P/S (Gibco-BRL). Then, 50% of the medium was replaced every two days.

### Establishment of Neuron–Astrocyte Cocultures

**FIGURE 1:**
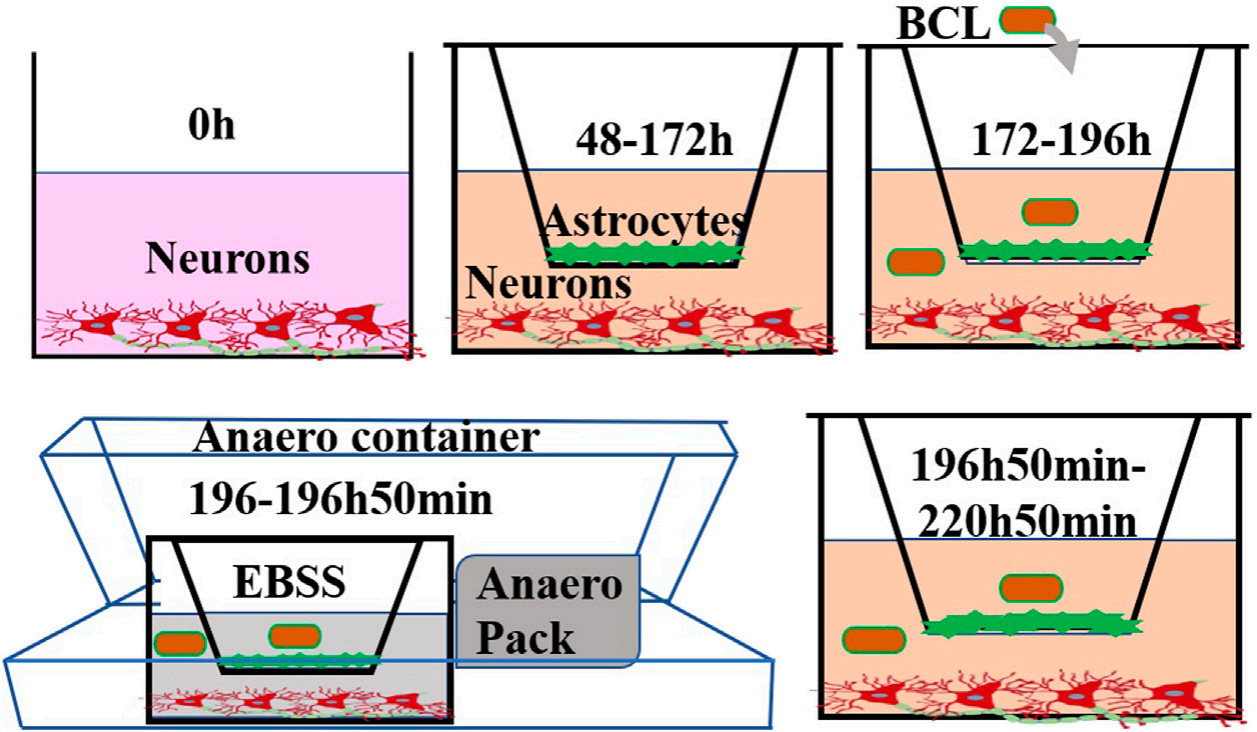
Experimental steps of this study. Neurons were cultured at the bottom of a Transwell filter at 0 h. After 48 h, astrocytes were seeded on the upper side of the inserts. At 172 h, the experiments were performed. The cells were treated with BCL for 24 h prior to OGD/R. To mimic ischaemic conditions, cocultures were subjected to OGD for 50 min, and the cultures were returned to a normoxic incubator for 24 h. Structural and functional evaluations under pathophysiological conditions were undertaken at 220 h 50 min.

Before starting the coculture, neurons were cultured on the bottom of a Transwell plate (Corning, 3460, 0.4 μm, New York, NY, United States), and the cultures were maintained in Neurobasal-A medium containing 2% B27, 0.25% GlutaMAX, and 1% P/S for at least 48 h. When the confluence gradually increased to 80%, astrocytes were used to establish the model. Astrocytes (2 × 10^5^ cells/cm^2^) were seeded into the matching well in the insert membrane in the Petri dish at 48 h. The time when the neurons were plated was defined as zero *in vitro*. At 172 h, the experiments were performed. The procedure for establishing the model is shown in Figure 1. The system for cocultivation according to the above method is defined as the NA system. Moreover, as controls, the cocultures were considered as the NA group, and neurons and astrocytes were cultured alone as the N group and A group, respectively.

### Protective Effects of Baicalin on Astrocytes and Neurons Exposed to Oxygen–Glucose Deprivation/Reoxygenation

The protective effects of BCL against the OGD/R insult in the NA model were evaluated at the most appropriate dose in different cell types. The neurons and astrocytes were seeded in 96-well plates. The cells were treated with different concentrations of BCL (137.5, 68.75, 34.38, 17.19, 8.59, 4.30, 2.15, and 1.08 μg/ml) for 24 h before OGD/R. The medium was replaced with deoxygenated Earle’s balanced salt solution without glucose with different concentrations of BCL. Subsequently, the cells were placed into the sealed Anaero container with an Anaero Pack (Mitsubishi, Tokyo, Japan) for 1 h. OGD was terminated by replacing Earle’s balanced salt solution (EBSS) with the complete medium with different concentrations of BCL, and the cells were cultured under normoxic conditions at 37°C for 24 h. Control indicates no treatment throughout the OGD/R in a standard medium. The cell viability was set as 100% in the control group. The model indicates treatment with OGD (1 h)/R (24 h).

### Oxygen–Glucose Deprivation/Reoxygenation Insult on NA and Drug Administration

As shown in Figure 1, we used OGD/R in the *in vitro* NA model to mimic ischemic conditions. Briefly, the medium was replaced with deoxygenated EBSS (Leagene Biotech Co., Beijing, China) at 196 h. Then, the NA models were placed into a sealed Anaero container with an Anaero Pack (Mitsubishi, Tokyo, Japan) for 50 min to initiate OGD insult. OGD was terminated by complete medium, and the cocultures were cultured under normoxic conditions at 37°C for 24 h to mimic reoxygenation. The cocultures with the above treatment were defined as the model group. The cocultures in the control group were incubated in DMEM/F12 with 20% FBS, 1% penicillin, and streptomycin without the above OGD/R treatment.

BCL was purchased from the National Institute for the Control of Pharmaceutical and Biological Products (Beijing, China). The BCL solution was prepared in glucose-free EBSS and DMEM/F12 containing 20% FBS and 1% P/S. The cultures were randomly divided into five groups: 1) control group, 2) OGD/R group, 3) BCL-L group (BCL: 8.59 μg/ml), 4) BCL-H group (BCL: 34.38 μg/ml), 5) BCL-H + ANA group (BCL + ANA12: 34.38 μg/ml+10 µM). The cells in the BCL-H group and BCL-L group were treated with BCL for 24 h prior to OGD/R and then under OGD conditions for 50 min. OGD was terminated, and the cells were cultured for an additional 24 h under normoxic conditions in the presence of BCL. The cocultures in the BCL-H and BCL-L groups were treated with BCL throughout the OGD/R process. The administration method of the BCL-H + ANA group was to add ANA-12 on the basis of BCL-H. Moreover, the N group, A group, and NA group treated with OGD/R were defined as the N + OGD/R group, A + OGD/R group, and NA + OGD/R group, respectively.

### Immunofluorescence and Image Acquisition

Immunofluorescence staining of neurons and astrocytes was performed. The neurons and astrocytes were fixed with 4% paraformaldehyde for 15 min. Then, the cells were blocked and permeabilized for 1 h using a mixture of 0.1% Triton X-100 (Fisher Scientific) and 10% goat serum (Sigma) in PBS. Finally, the cells were incubated with primary antibodies against MAP2 (Abcam) and GFAP (Abcam) at 4°C overnight. Cells were incubated with secondary antibodies (Alexa Fluor 647-conjugated goat anti-chicken IgY, Abcam; FITC-conjugated goat anti-mouse IgG, Abcam) for 1 h. Images were captured by Olympus fluorescence microscopy after staining the nuclei with DAPI (4′’,6-diamidino-2-phenylindole).

### Flow Cytometric Analysis of Apoptosis

Analysis of apoptosis was carried out by flow cytometry using an Annexin V Apoptosis Detection Kit. Cells were digested with EDTA-free enzymes, harvested and washed twice with PBS, and then resuspended in 500 µL binding buffer. Next, the cells were stained with 5 μL Annexin V FITC and 5 μL PI (Kegen, Nanjing, Jiangsu Province, China), and the resuspended cells were incubated for 10 min in the dark. Finally, the cells were immediately examined on a FACSCalibur (BD Biosciences, Franklin, NJ, United States) flow cytometer, and the data were analyzed with Cell Quest software (BD Biosciences).

### Assay of Inflammation and Oxidative Stress Activity

Interleukin-1β (IL-1β; Wuhan Liu he Biotechnology Co., Wuhan, China), interleukin-6 (IL-6; Wuhan Liu he Biotechnology Co.), and tumor necrosis factor-α (TNF-α; Proteintech) were assessed using ELISA kits according to the manufacturer’s instructions. The optical density was measured at a wavelength of 450 nm using a microplate reader (Biotek, Winooski, Vermont, United States). Malondialdehyde (MDA; Jiancheng, Nanjing, China), superoxide dismutase (SOD; Jiancheng), and nitric oxide (NO; Jiancheng) were measured using commercial kits according to the manufacturer’s instructions.

### Assay of Brain-Derived Neurotrophic Factor by ELISA

The neurons cultured alone, astrocytes cultured alone, and neuron–astrocyte coculture system were treated with OGD/R. BCL was administered to the NA system after OGD/R. Their supernatants were harvested to determine the levels of the secreted BDNF. Additionally, cytoplasm contents in neurons or astrocytes in the NA + OGD/R group, NA + BCL-H + OGD/R group, and NA group were obtained using lysis buffer containing 50 mM Tris-HCl PH7.4, 2% SDS, 0.1 M NaCl, 1 mM EDTA, 1% Triton X-100, 0.5 μg/ml aprotinin, 1 mM sodium orthovanadate, and 1 mM PMSF. The levels of BDNF (Wuhan Liu he Biotechnology Co.) were measured by ELISA according to the manufacturer’s instructions. The optical density was measured at a wavelength of 450 nm using a microplate reader (Biotek, Winooski, Vermont, United States).

### Western Blot Analysis

The changes in protein levels were quantified using WB analysis. Cells were washed with PBS. Protein was obtained using the RIPA buffer (PPLYGEN, China) according to the manufacturer’s instructions. The resuspended supernatant was quantified for the protein concentration using the BCA protein assay kit (KeyGen). Protein samples (30 mg per sample) were resolved using a 10% Tris/glycine SDS-PAGE gel and then transferred to a polyvinylidene difluoride membrane. Membranes were incubated in PBST containing 5% nonfat milk for 30 min. Following incubation with the primary antibodies in PBST/TBST containing 5% BSA that recognize anti–microtubule-associated protein-2 (MAP-2, Abcam), anti-cysteinyl aspartate-specific proteinase-3 (caspase-3, Proteintech), anti-cysteinyl aspartate-specific proteinase-9 (caspase-9, Proteintech), anti–B-cell lymphoma 2 (Bcl-2, Affinity), anti–Bcl-2 associated X protein (Bax, Proteintech), anti-TrkB (Affinity), anti–p-TrkB (Affinity), anti-CREB (Affinity), anti–p-CREB (Affinity), anti-PI3K (Affinity), anti–p-PI3K (Affinity), anti-Akt (Affinity), anti–p-Akt (Affinity), anti–mitogen-activated protein kinase (anti-MAPK (Affinity), anti–p-MAPK (Affinity), anti-extracellular signal-regulated kinase1/2 (anti-ERK1/2, Affinity), anti-CREB (Affinity), anti–p-ERK1/2 (Affinity), and anti-GAPDH at 4°C overnight, the blots were washed and then incubated with anti-rabbit IgG for 1 h at 25°C. After subsequent washes in TBST, immunolabeling was detected using enhanced chemiluminescence reagents (Perkin Elmer, Waltham, MA) and quantified by densitometry using an image analyzer (Bio-Rad, United States).

### Statistical Analysis

All data are expressed as the mean ± standard deviation. Multiple comparisons were performed using one-way analysis of variance (ANOVA), and each group of data was subjected to the least significant difference (LSD) test using SPSS 20.0 (SPSS, Chicago, IL, United States). An unpaired two-tailed *t*-test was performed for comparisons between two groups. Values of *p* < 0.05 were considered to be significant, and *p* < 0.01 was considered to be highly significant.

## Results

### Neuronal Morphology in N Group and NA Group

**FIGURE 2:**
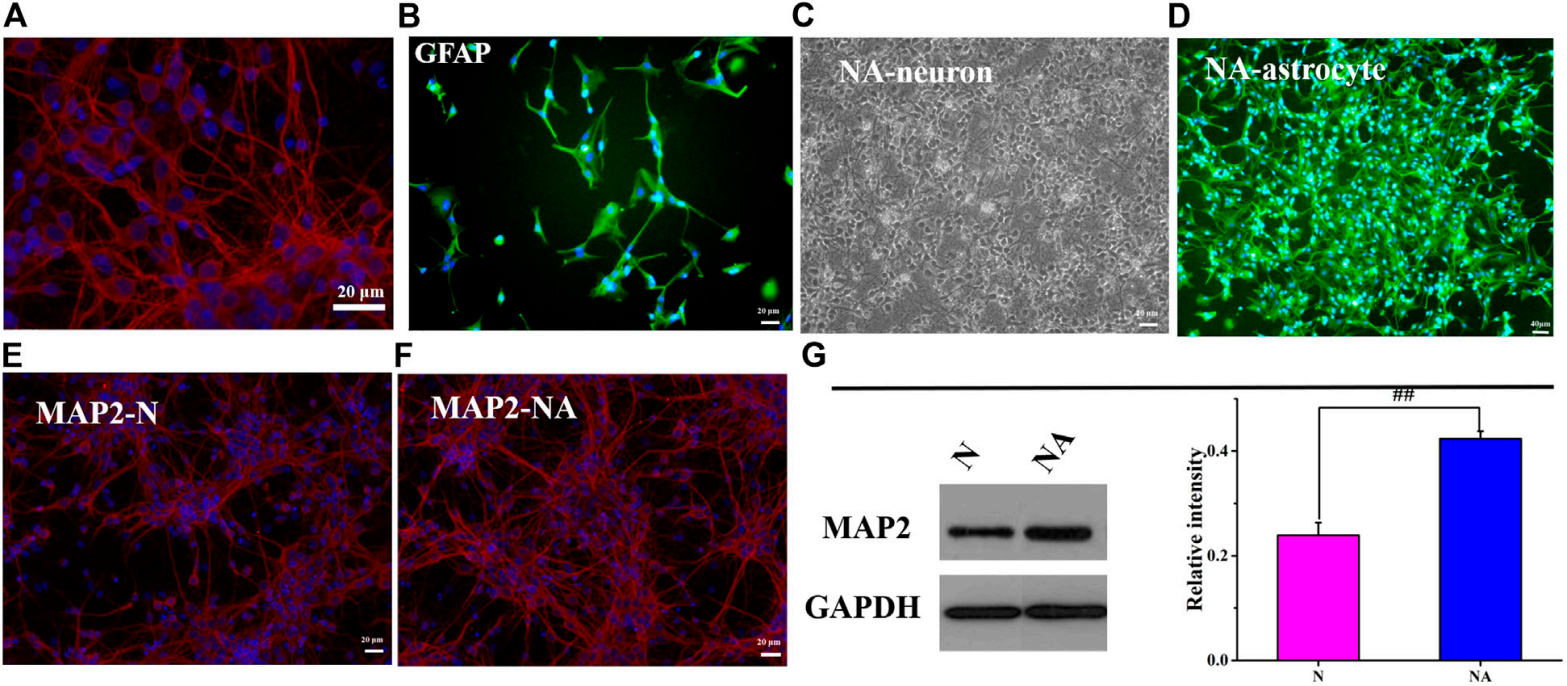
Neuronal morphology in the N group and NA system. **(A)** Neurons were positive for MAP-2 (red). **(B)** Astrocytes were positive for GFAP (green). **(C)** The morphology of neurons in the coculture system. **(D)** The morphology of astrocytes in the coculture system. **(E)** MAP-2 (red) in the N group (N). **(F)** MAP-2 (red) in the NA group (NA). **(G)** The expression of MAP2 in the N group and NA group. ##*p* < 0.01, N vs. the NA group.

The purity of neurons and astrocytes was in line with that in a previous study (Li et al., 2019b; Li et al., 2019a) (Figures 2A,B). As shown in Figures 2C,D, we observed the growth conditions of neurons and astrocytes in the coculture system. To better observe the morphology of the neurons, we compared the morphology of neurons in the absence or presence of astrocytes at 172 h. MAP2 is a marker of neuron cell bodies, dendrites, and dendritic spines (Zhu et al., 2016). Accordingly, we performed immunofluorescence staining of MAP2 in neurons. As expected, neurons cocultured with astrocytes were significantly more mature than neurons cultured alone. As shown in Figures 2E,F, the number of neurons in the coculture system was greater than that in the monoculture system. Neuronal synapses were more fully extended, elongated, branched, and formed a distinct network with an increased number of branches in the coculture systems. Moreover, WB results showed that the MAP2 level was significantly higher (*p* < 0.01) in the NA system than that in the monoculture cocultured with astrocytes (Figure 2G). The results indicated that astrocytes had promoting effects on neurite outgrowth and neuronal network formation. The *in vitro* coculture system was a more reliable, scalable, and suitable tool to study accurately the aspects of astrocyte–neuron functional properties and interactions while being easily accessible for cell type–specific manipulations and observations.

### The Damage of Neurons Exposed to Oxygen–Glucose Deprivation/Reoxygenation

**FIGURE 3:**
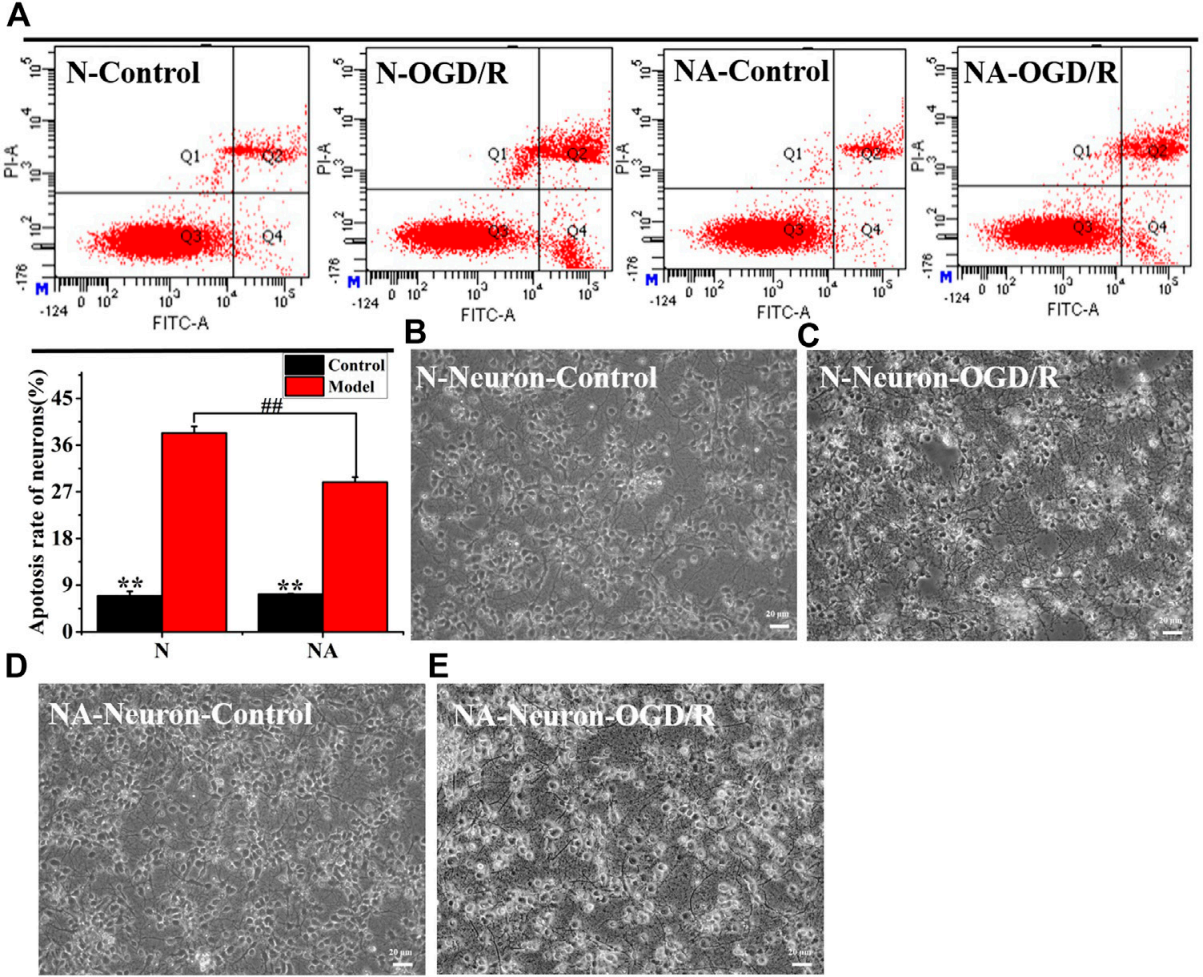
Neuronal apoptosis and morphology in N and NA groups. **(A)** Neuron apoptosis in the N and NA groups. **(B)** Neuronal morphology in the N group. **(C)** Neuronal morphology in the N group after OGD/R. **(D)** Neuronal morphology in the NA group. **(E)** Neuronal morphology in the NA group after OGD/R. **p < 0.01, vs. the model group; ##*p* < 0.01, N vs. the NA group.

We evaluated neuronal apoptosis and morphology in different culture systems after OGD to observe the effects of astrocytes on neurons (Figures 3A–E). The results showed that OGD/R induced significant neuronal apoptosis (Figure 3A). However, in comparison with cultures in the absence of astrocytes, the coculture system led to dramatically less neuronal apoptosis (*p* < 0.01) (Figure 3A). In the neuron system cultured alone, the neurons fell off, and the axons of the neurons were severely broken after OGD/R (Figure 3C). The morphology of neurons in the NA coculture system is better than that in the N group (Figures 3C,E). The results indicated that astrocytes exerted a beneficial protective effect on neurons after OGD/R.

### Protective Effects of Baicalin on Astrocytes and Neurons Exposed to Oxygen–Glucose Deprivation/Reoxygenation

**FIGURE 4:**
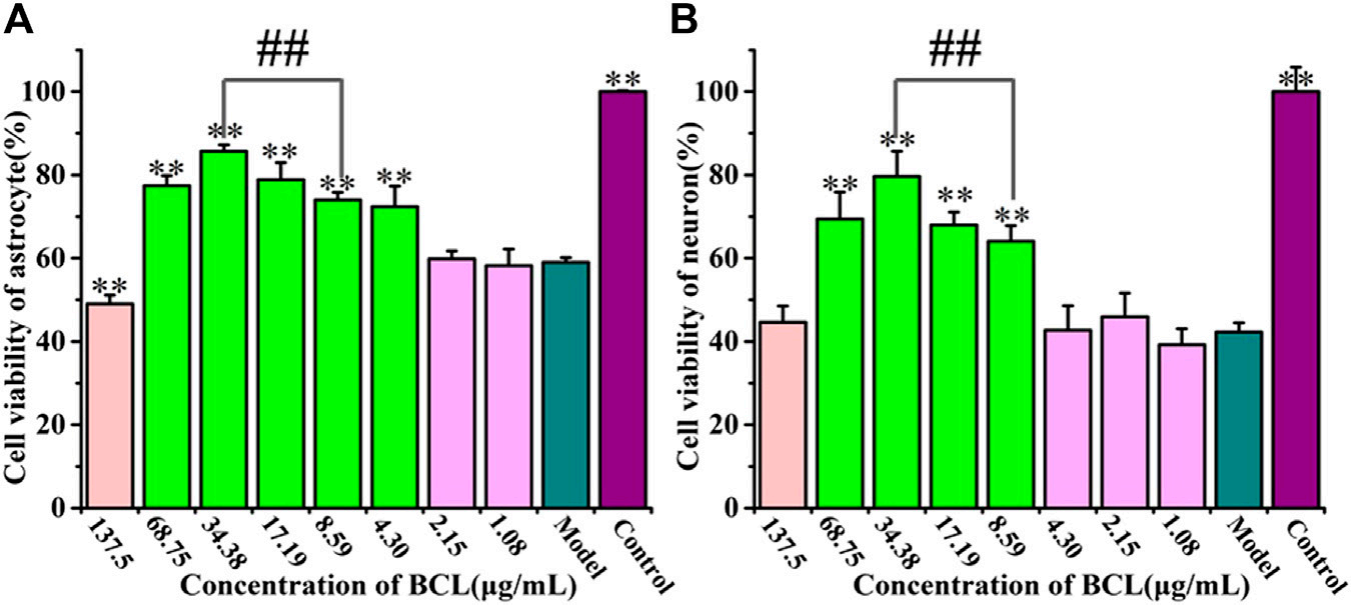
Protective effects of baicalin on astrocytes and neurons after OGD/R. **(A)** Cell viability counts indicating the protective effects of BCL on astrocytes after OGD/R. **(B)** Cell viability counts showing the protective effects of BCL on neurons after OGD/R. **p < 0.01, vs. the model group; ##*p* < 0.01, 34.38 μg/ml vs. 8.59 μg/ml.

As shown in Figure 4, BCL was not toxic at the concentration of 8.59–68.75 μg/ml and showed significant protective effects in this study. We found that 34.38 μg/ml BCL was the optimal dose and showed a more significant protective effect than 8.59 μg/ml BCL (*p* < 0.01).

### Reactive Astrocytes and Brain-Derived Neurotrophic Factor Secretion after Oxygen–Glucose Deprivation/Reoxygenation

**FIGURE 5:**
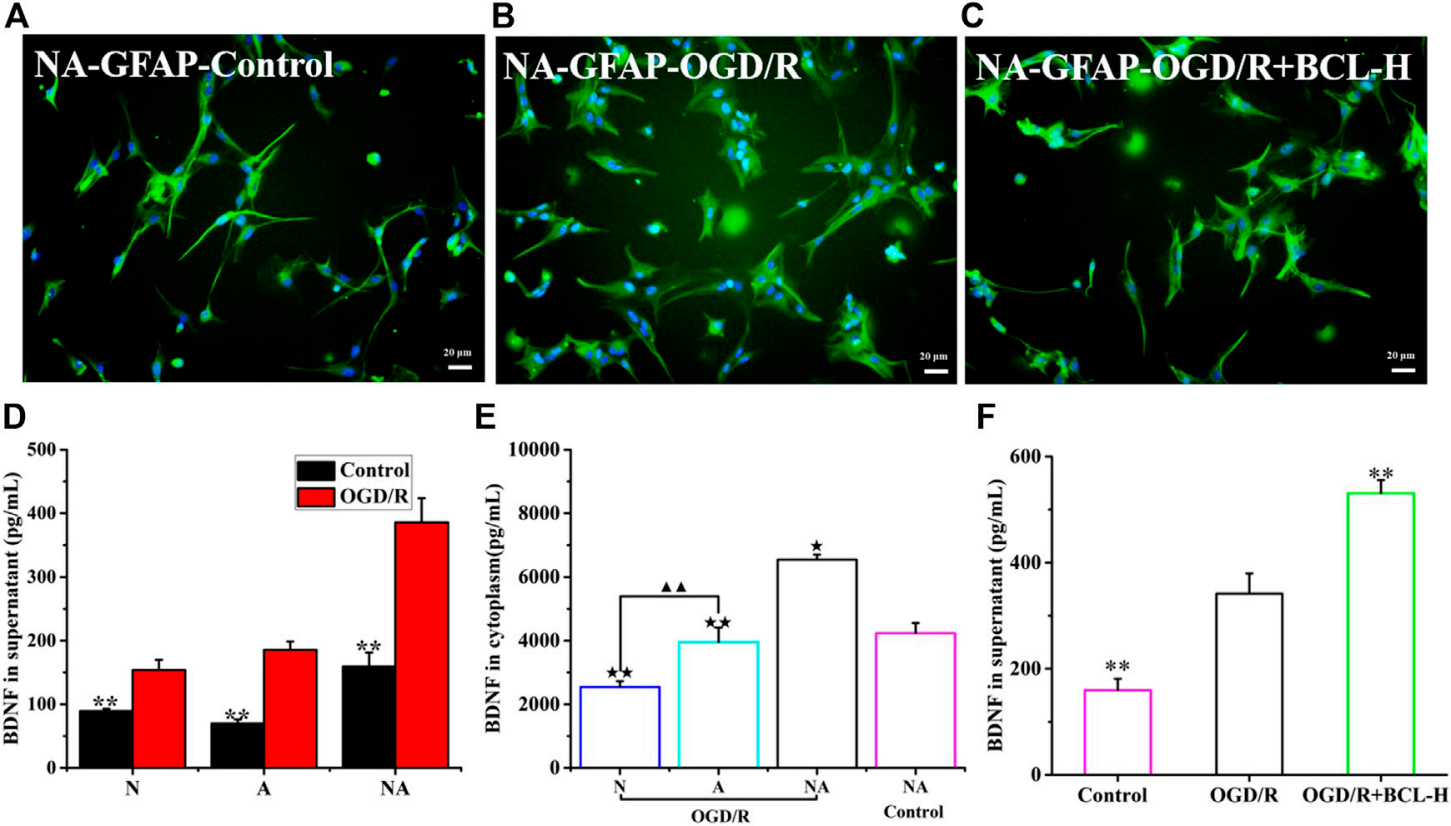
Reactive astrocytes and BDNF secretion after OGD/R. **(A)** Morphology of astrocytes (GFAP) in the NA coculture system in the control group. **(B)** Morphology of astrocytes (GFAP) in the NA coculture system after OGD/R. **(C)** The effects of BCL on the morphology of astrocytes in the NA coculture system after OGD/R. **(D)** BDNF release in the N group, A group, and NA group after OGD/R. **(E)** The level of BDNF in neuronal cytoplasm (N), astrocyte cytoplasm (A), and NA system. **(F)** The effects of BCL on BDNF release in the NA group after OGD/R. ★★*p* < 0.01, ★*p* < 0.05 vs. NA + BCL-H + OGD/R group; ▲▲*p* < 0.01, N OGD/R group vs. A OGD/R group; **p < 0.01, vs. OGD/R group.

A previous study showed that astrocytes are activated and they undergo important morphological changes to become reactive astrocytes after ischemia (Choudhury and Ding, 2016). In addition to the detrimental role during the early stage of ischemic onset, reactive astrocytes play a beneficial role in the brain (Liu and Chopp 2016). In view of the protective effect of astrocytes on neurons, we observed changes in astrocytes in the coculture system. Intermediate filament GFAP is a marker of astrocyte activation, which is essential for the cell structure and cell function of astrocytes (Choudhury and Ding, 2016). Previous studies have shown that astrocytes around the lesion were reactivated and that GFAP was dramatically upregulated in a time- and space-dependent manner after IS (Choudhury and Ding 2016). Our research results were in line with those of previous theories. We observed the morphological structure of astrocytes, which had a polygonal morphology, more synapses, and larger cell bodies after OGD/R using immunofluorescence by detecting the expression of GFAP, was very in line with the characteristics of reactive astrocytes (Figure 5B). Astrocytes expressed high levels of GFAP after OGD/R, while the number of astrocytes was not increased, which was different from the control astrocytes (Figures 5A,B). Activated reactive astrocytes protect neurons after stroke by producing a variety of neurotrophic factors, including nerve growth factor, basic fibroblast growth factor, BDNF, and glial cell–derived neurotrophic factor (Liu and Chopp 2016). BDNF, the most abundant neurotrophin in the mammalian CNS, exerts neuroprotective effects in ischemic stroke (Liu et al., 2020). Accumulating studies have suggested that BDNF was produced in neurons, but astrocytes were also an important source of BDNF in the brain (Hong et al., 2016). BDNF is released from astrocytes *via* exocytosis to regulate the function of neighboring neurons (Nobukazu et al., 2015; Hong et al., 2016). Based on this OGD/R model, very few studies have focused on the release of BDNF from neurons or astrocytes and the coculture system of neurons and astrocytes. Therefore, we detected the level of BDNF in the A group, N group, and NA group after OGD/R. Apart from neurons, astrocytes cultured alone can also produce BDNF after OGD/R. The levels of BDNF were higher in the NA system than those in neurons or astrocytes cultured alone (*p* < 0.01) (Figure 5D). Interestingly, the content of BDNF in the coculture system was higher than the sum of neurons and astrocytes cultured separately (*p* < 0.01). We further detected the content of BDNF in the cytoplasm of neurons and astrocytes in the NA culture system after OGD/R. We found that BDNF in the cytoplasm of astrocytes was significantly higher than that in neurons (*p* < 0.01) (Figure 5E). It is speculated that the reason is that the neurons were severely damaged and the astrocytes were in an activated state to become the important source of BDNF. To examine whether the activated state of astrocytes can be maintained by BCL, we measured the morphology of astrocytes treated with BCL after OGD/R. Without increasing the number of astrocytes, BCL maintained the activated state of astrocytes (Figure 5C). Moreover, we wanted to investigate whether BCL can affect the synthesis and secretion of BDNF. The results showed that BDNF was secreted more in the model group than in the control group (*p* < 0.01), and BCL treatment further promoted this progress (Figure 5F). To further observe the source of BDNF, we detected the content of BDNF in the cytoplasm. The results showed that BDNF was synthesized more in the A + OGD/R group than in the N + OGD/R group (*p* < 0.01) (Figure 5E).

### The Effects of Baicalin on the Expression of TrkB and CREB in the NA System after Oxygen–Glucose Deprivation/Reoxygenation

**FIGURE 6:**
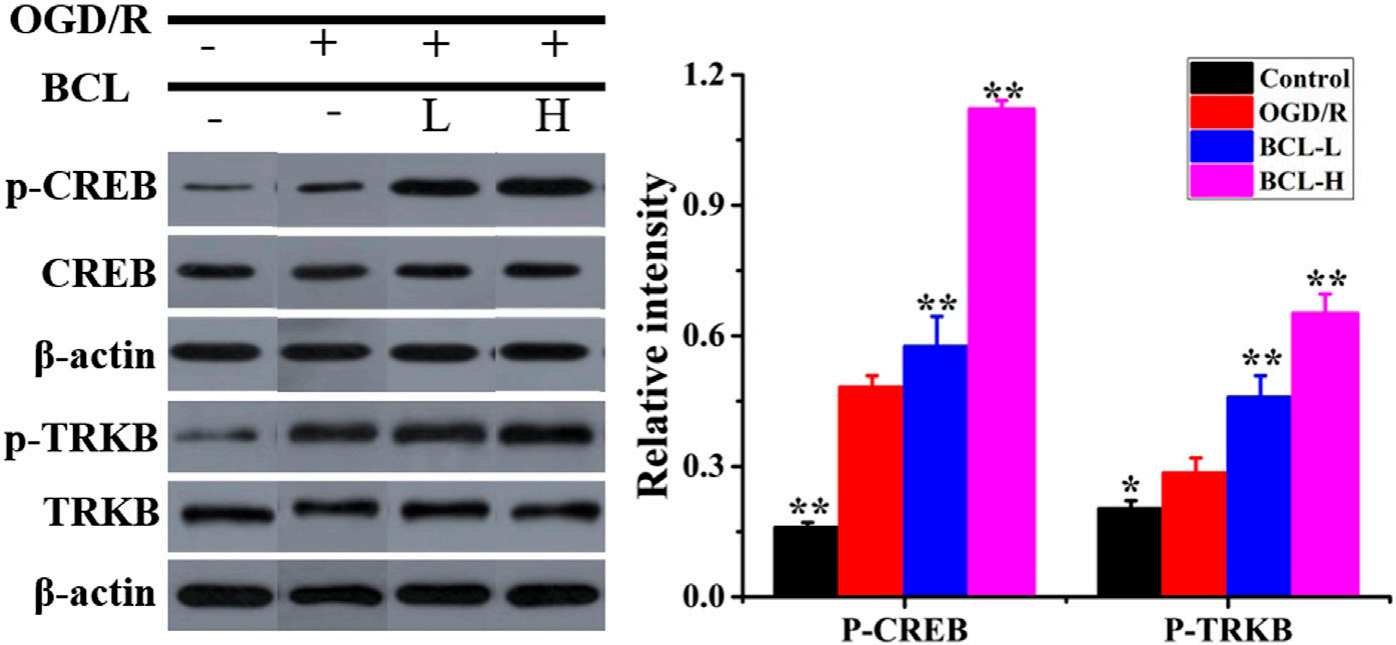
Effects of BCL (34.38 μg/ml, 8.59 μg/ml) on the expression of CREB and TrkB in the NA system after OGD/R. Quantified results were normalized to β-actin expression. **p < 0.01, *p < 0.01, vs. OGD/R group.

Considering the promoting effect of BCL on the synthesis and secretion of BDNF, we examined whether BCL treatment could promote the expression of CREB, a transcription factor that increased BDNF expression (Bo et al., 2015). WB assays were performed for p-CREB with BCL treatment in the NA system after OGD/R. The data (Figure 6) showed that the expression of p-CREB in the OGD/R group was significantly higher than that in the control group (*p* < 0.01). BCL treatment upregulated the expression of p-CREB (*p* < 0.01). Activated CREB initiates the transcription of BDNF genes. Mature BDNF binds with higher-affinity Trk receptors to increase axonal growth, differentiation, morphology, neuronal survival, synaptic plasticity, and the resultant networks. Therefore, we examined the expression of TrkB. As shown in Figure 6, TrkB was expressed more in the model group than in the control group, and BCL treatment further promoted this progress (*p* < 0.01).

### Baicalin Maintained the Morphology of Neurons and the Expression of MAP2 after Oxygen–Glucose Deprivation/Reoxygenation

**FIGURE 7:**
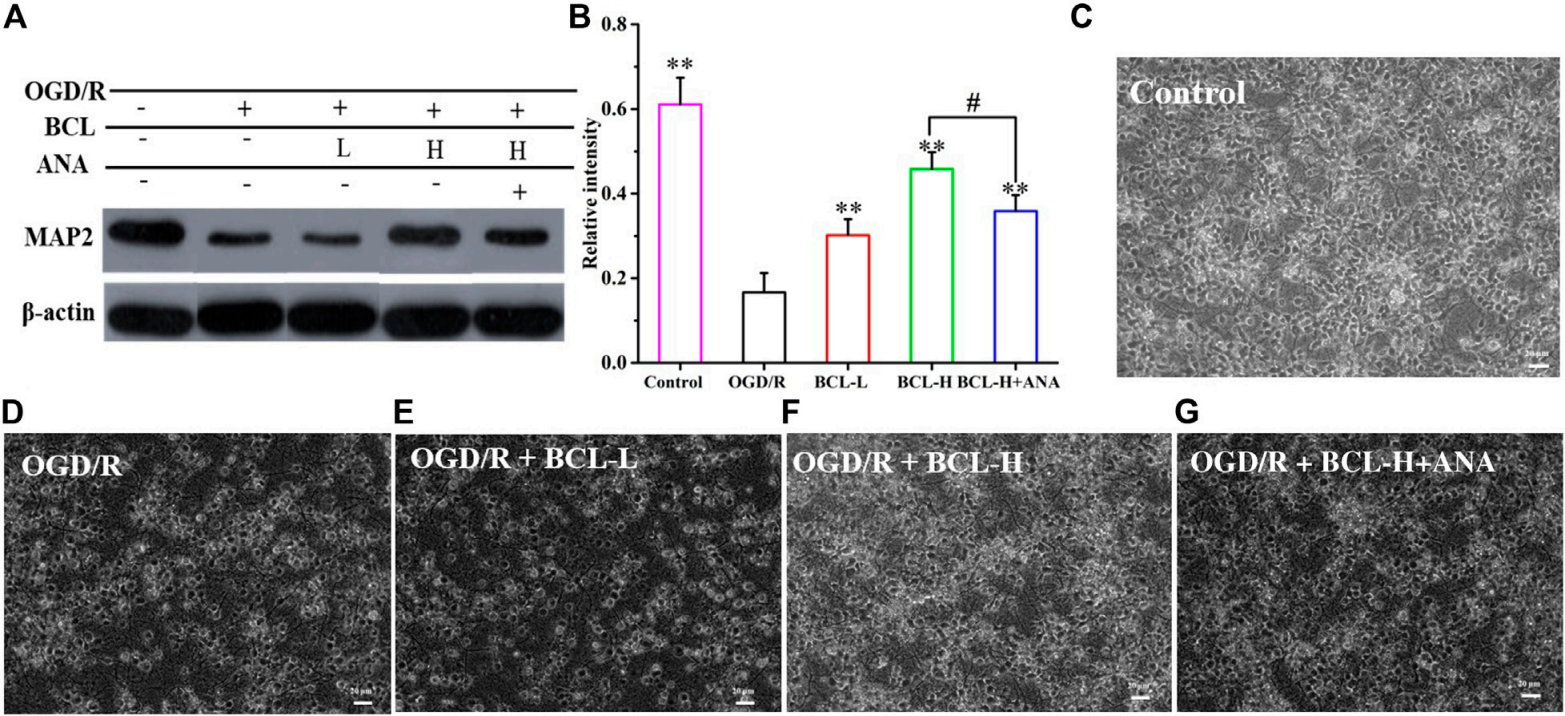
Effects of BCL (34.38 μg/ml and 8.59 μg/ml) and TrkB antagonist on neurons in the NA system after OGD/R. **(A–B)** The effect of BCL on the expression of MAP2. **(C)** Neuronal morphology in the control group. **(D)** Neuronal morphology in the OGD/R group. **(E)** Neuronal morphology in the OGD/R + BCL-L group. **(F)** Neuronal morphology in the OGD/R + BCL-H group. **(G)** Neuronal morphology in the OGD/R + BCL-H + ANA group. Quantified results were normalized to β-actin expression. **p < 0.01, vs. OGD/R group, #*p* < 0.05, BCL-H group vs. BCL-H+ANA group.

MAP2, a neuron-specific cytoskeletal protein, is an independent criterion for defining axons that reflect neuronal morphology (Zhang et al., 2019). As shown in Figures 7A,B, MAP2 expression was significantly lower in the model group than that in the control group (*p* < 0.01), while the BCL group demonstrated higher MAP2 expression than that in the model group (*p* < 0.01). Compared with the model group, MAP2 expression was increased in the treatment groups (*p* < 0.01). In addition to WB, we performed microscopic imaging to observe the morphological changes in neurons. We found that neuronal axons were fully extended and formed a distinct network in the control group (Figure 7C). OGD/R-treated neurons displayed morphological collapse with significantly destructive dendrites and shedding of cells (Figure 7D). However, BCL treatment prevented the occurrence of morphological collapse upon OGD/R insult (Figures 7E,F). To confirm the hypothesis that the BDNF-TrkB pathway mediates the protective effect of BCL against OGD/R-induced injury, we next explored whether ANA12 reverses the protective effect of BCL against OGD/R-induced injury in neurons. Interestingly, inhibition of TrkB with ANA12 partially blocked the neuroprotective effects of BCL (Figures 7A,B,G). These results strongly suggested that BDNF-TrkB signaling played an essential role in BCL-mediated neuroprotective effects against OGD/R-induced damage.

### Baicalin Attenuated Oxygen–Glucose Deprivation/Reoxygenation–Induced Apoptosis

**FIGURE 8:**
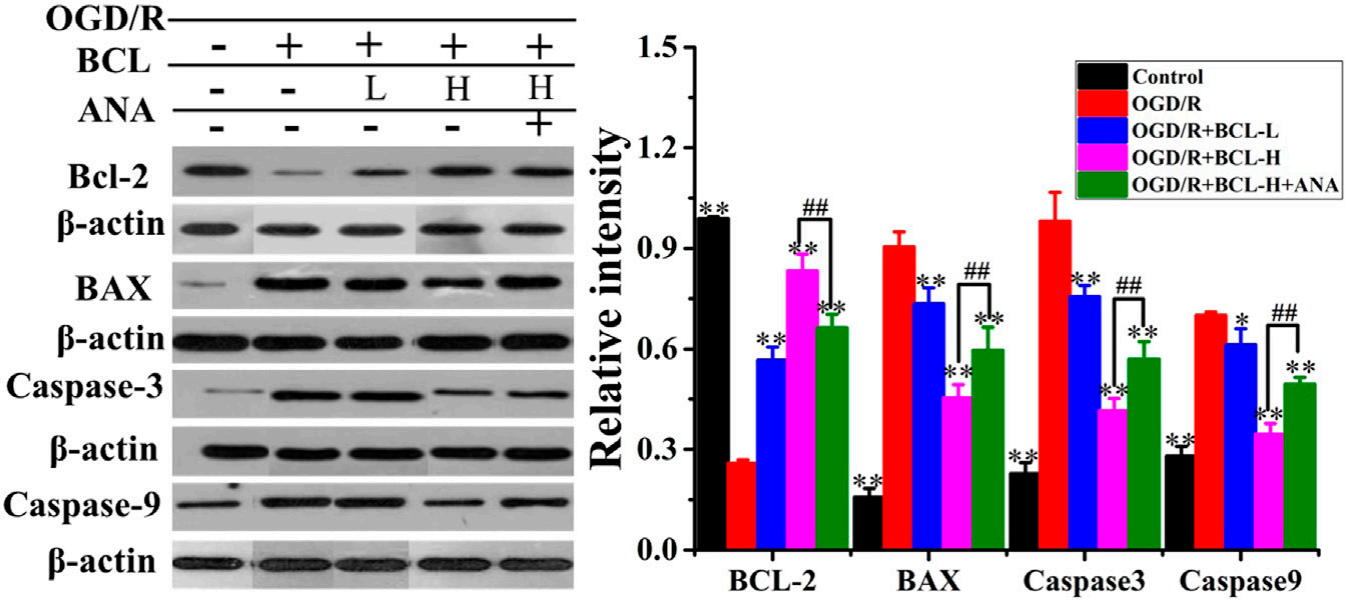
Effect of BCL (34.38 μg/ml and 8.59 μg/ml) on apoptosis proteins after OGD/R. Quantified results were normalized to β-actin expression. **p < 0.01, *p < 0.05, vs. model group, ##*p* < 0.01, OGD/R + BCL-H group vs. OGD/R + BCL-H + ANA group.

WB was performed to confirm the effect of BCL in preventing OGD/R-induced apoptosis in neurons, and we found that OGD/R significantly induced neural apoptosis (Figure 8). However, relative to that of the OGD/R group, BCL treatment effectively attenuated OGD/R-induced apoptosis. Nevertheless, the apoptosis inhibitory effect of BCL in neurons can be attenuated by ANA12, a potent and selective TrkB antagonist that suppresses the activity of TrkB and its downstream signaling axis. As shown in Figure 8, Bcl-2 expression was significantly lower in the OGD/R group than that in the control group (*p* < 0.01), while the BCL group demonstrated higher Bcl-2 expression than that in the OGD/R group (*p* < 0.01). However, in contrast to Bcl-2 expression, Bax, caspase-3, and caspase-9 expressions were significantly higher in the model group than those in the control group (*p* < 0.01), while lower Bax, caspase-3, and caspase-9 expressions were observed in the treatment groups than those in the model group (*p* < 0.01). These effects of BCL on Bcl-2 expression were suppressed by treatment with ANA12; in contrast, Bax, caspase-3, and caspase-9 protein levels were increased (*p* < 0.01). From the aforementioned results, we concluded that BCL was effective in preventing OGD-induced apoptosis to protect neurons. BDNF-TrkB signaling was necessary for the neuroprotective actions of BCL on neurons in the OGD/R-treated NA coculture system. The neuroprotective actions of BCL in the NA coculture system were mediated at least in part by BDNF.

### The Effects of Baicalin and ANA12 on Inflammation and Free Radicals after Oxygen–Glucose Deprivation/Reoxygenation

**FIGURE 9:**
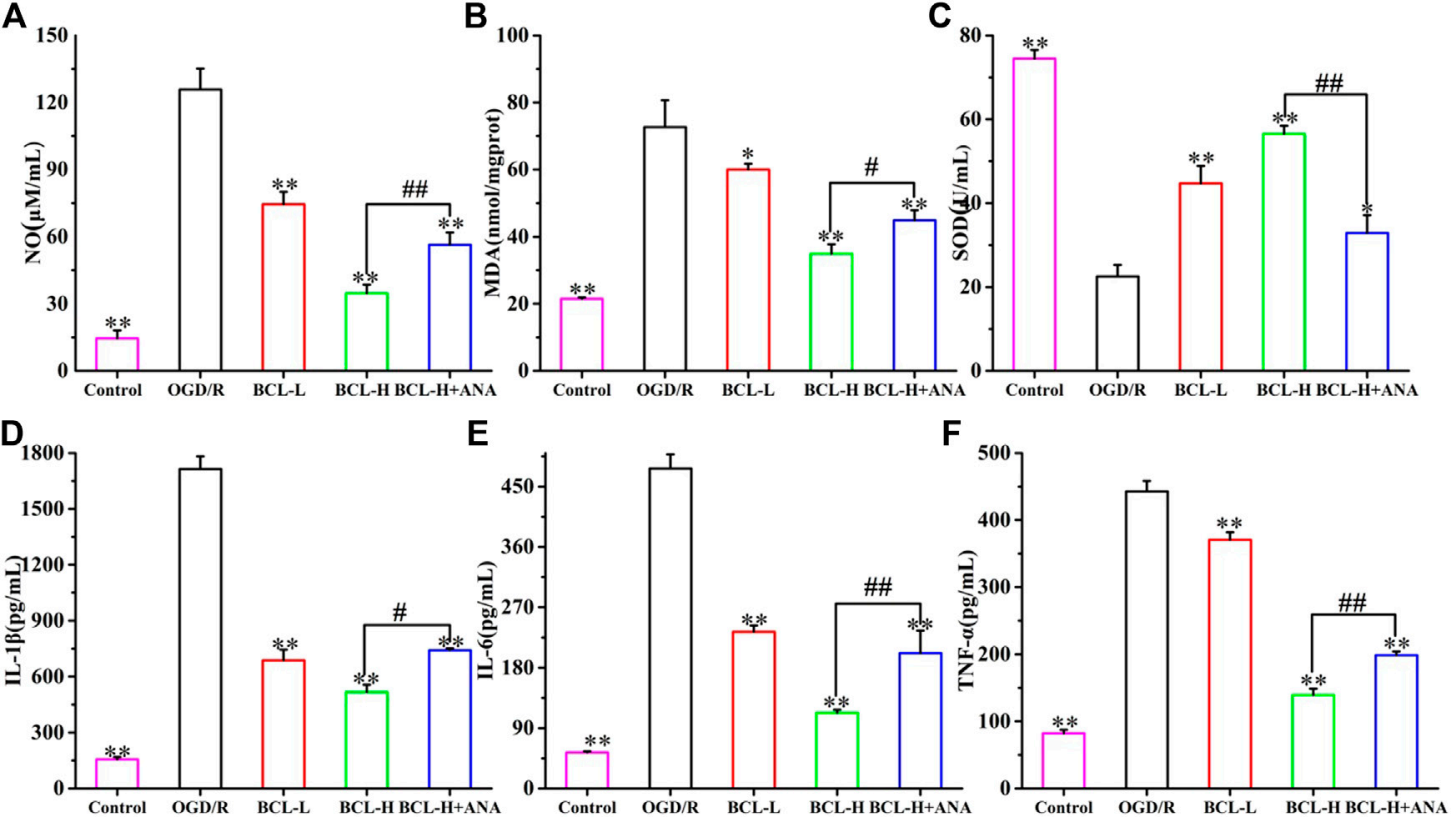
Effect of BCL (34.38 μg/ml, 8.59 μg/ml) on inflammation and oxidative/nitrosative stress in the NA system after OGD/R. **(A)** The effect of BCL on NO in the NA system after OGD/R. **(B)** The effect of BCL on MDA in the NA system after OGD/R. **(C)** The effect of BCL on SOD in the NA system after OGD/R. **(D)** The effect of BCL on the IL-1β level in the NA system after OGD/R. **(E)** The effect of BCL on IL-6 in the NA system after OGD/R. **(F)** The effect of BCL on TNF-α in the NA system after OGD/R. **p < 0.01, *p < 0.05, vs. OGD/R group; #*p* < 0.05, ##*p* < 0.01, BCL-H group vs. BCL-H+ANA group.

The brain represents almost 20% of the body’s oxygen consumption under normal physiological conditions, generating more free radicals than other organs. After the ischemia-reperfusion injury, the brain produces a large number of free radicals, mainly oxidative nitrosative stress, which plays an important role in the pathogenesis mechanism of IS (Sun et al., 2018). However, the characteristics of the brain are more vulnerable to free radical damage. Moreover, inflammation, a secondary injury mechanism following IS, plays an important role in the progression of IS. Inhibition of inflammation and oxidative/nitrosative stress can decrease brain injury and improve neurological outcomes (Yenari et al., 2014). The results of the present study are in line with the above theory. As shown in Figures 9A–C, compared with the control group, the expression of NO (Figure 9A) and MDA (Figure 9B) after OGD/R increased, and SOD (Figure 9C) significantly decreased (*p* < 0.01). However, the elevation of NO and MDA was significantly attenuated by BCL treatment. BCL treatment increased the expression level of SOD (*p* < 0.01). Furthermore, we evaluated the effect of BCL on the expression of IL-1β (Figure 9D) and IL-6 (Figure 9E) and TNF-α (Figure 9F). Compared with the control group, OGD/R treatment significantly increased the expression levels of TNF-α, IL-1β, and IL-6 (*p* < 0.01). Compared with that in the OGD/R group, BCL robustly reduced the release of inflammatory cytokines (*p* < 0.01). The results indicate that BCL has a dose-dependent effect against oxidative/nitrosative stress and inflammation. To verify further whether TrkB activation was required for the neuroprotective effect of BCL, we examined the effect of ANA12, an TrkB Receptor antagonists, on inflammation and free radicals induced by OGD/R. In the presence of ANA12, compared with those of the BCL-H group, the effects of BCL on oxidative/nitrosative stress and anti-inflammatory effects of BCL were significantly reduced (NO, SOD, IL-6, and TNF-α: *p* < 0.01; MDA and IL-1β: *p* < 0.05). These experiments demonstrated that BDNF-TrkB signaling was necessary for the neuroprotective actions of BCL in the OGD/R-treated NA coculture system. Our findings here indicated that the neuroprotective actions of BCL in the NA coculture system were mediated at least in part by BDNF.

### The Effects of Baicalin and TrkB Receptor Antagonists on the Expression of PI3K and Akt in the NA System after Oxygen–Glucose Deprivation/Reoxygenation

**FIGURE 10:**
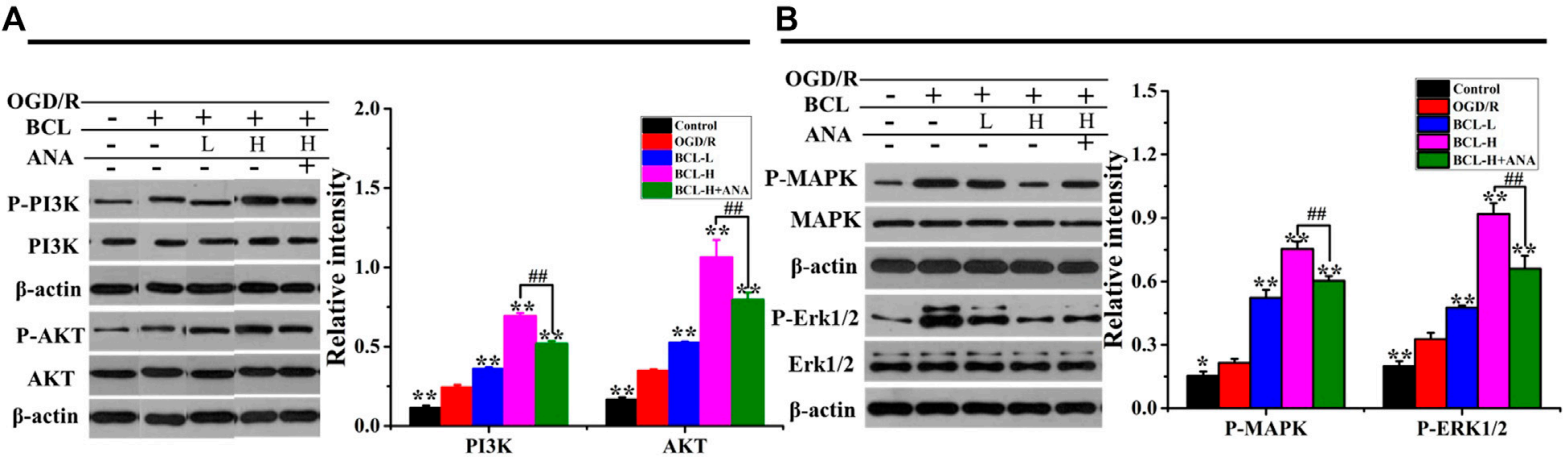
Effects of BCL (34.38 μg/ml and 8.59 μg/ml) and ANA12 on the expression of PI3K, Akt, MAPK, and ERK in NA systems after OGD/R. **(A)** The effect of BCL on the expression of PI3K and Akt in the NA system after OGD/R. **(B)** The effect of BCL on the expression of MAPK and ERK in NA systems after OGD/R. Quantified results were normalized to β-actin expression. **p < 0.01, vs. OGD/R group; ##*p* < 0.01, BCL-H group vs. BCL-H + ANA group.

The expression of BDNF and the moderate activity of its associated signaling axes, such as TrkB/PI3K/Akt and TrkB/MAPK/ERK1/2, are required for neuronal survival, neuron morphology, anti-inflammation, antioxidant stress, and antiapoptosis. Therefore, it is interesting to propose that BCL mediated neuroprotection is mediated by modulating BDNF/TrkB and their downstream signaling pathways. BDNF binding to high-affinity TrkB triggers its dimerization and autophosphorylation of intracellular tyrosine residues, leading to downstream PI3K/Akt signaling activation (Kowianski et al., 2018). The PI3K/Akt signaling pathway exerts multiple cellular functions, including antiapoptotic and pro-survival activities, enhancement of dendritic growth and branching, and long-term maintenance of synaptic plasticity (Yoshii and Constantine-Paton 2010; Kowianski et al., 2018; Liu et al., 2020). The MAPK/ERK signaling pathway plays a critical role in cell growth and differentiation, protein synthesis–dependent plasticity, neuronal survival, and neuronal differentiation promotion (Autry and Monteggia 2012; Kowianski et al., 2018). As shown in Figure 10, we found that the model group had significantly higher expressions of PI3K, Akt, MAPK, and ERK after OGD/R than the control group in the NA system (*p* < 0.01). BCL treatment significantly promoted the expression of PI3K, Akt, MAPK, and ERK (*p* < 0.01). Moreover, statistical analysis of the data indicated that the BCL-H group had significantly higher expressions of PI3K, Akt, MAPK, and ERK in the NA system after OGD/R than the BCL-H + ANA12 group (*p* < 0.01); that was, the effects of BCL were suppressed by pretreatment with ANA12.

## Discussion

Previous studies have often focused on the changes and plasticity of neurons in the recovery of neurological function after IS. However, other cells with complex specific signals and cascade reactions that affect neuronal recovery also need to be studied. Interestingly, the pharmacological agents tested to target neurons may also affect other neuronal functions. As a major constituent of the central nervous system, astrocytes constitute 50% of all the cells in the central nervous system and play essential roles in maintaining brain function (Filous and Silver 2016). In particular, astrocytes contribute to neurogenesis and synaptogenesis in normal brain function and axonal remodeling after IS (Liu and Chopp 2016). In light of the many actions of astrocytes on neurons, drugs targeting reactive astrocytes may effectively sustain neuronal function and hence survival after IS. Constructing an *in vitro* neuron and astrocyte coculture system can provide an advantageous platform for studying the interaction between astrocytes and neurons and the therapeutic effects of drugs on neurons through astrocytes. In line with a previous study, we found that astrocytes provided positive protection to neurons under normal and pathological conditions. In the presence of astrocytes, the neuronal phenotype was more neuroprotective effect of BCL the characteristics of brain tissue *in vivo*. After OGD/R, the morphology of neurons in the coculture system was better, and the apoptosis rate was lower. Astrocytes become reactive in response to pathological conditions following IS. Our research was consistent with previous research theories. Compared with the control group, the morphology of astrocytes conformed to the characteristics of reactive astrocytes, which had a polygonal morphology, larger cell bodies, and an increased number of synapses after OGD/R. A previous study showed that reactive astrocytes played an important role in neuroprotection and neurorestoration after IS.

**FIGURE 11:**
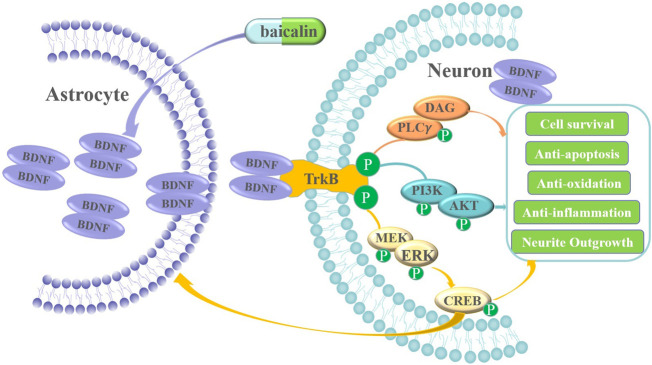
Scheme of the neuroprotective mechanism of BCL after OGD/R.

The release of neurotrophic factors is an important reason for the protective effect of reactive astrocytes (Sofroniew 2005; Choudhury and Ding 2016). BDNF is released from astrocytes *via* exocytosis to regulate the function of neighboring neurons (Zhang et al., 2015; Hong et al., 2016; Shih et al., 2017). For the first time, our research focused on the release of BDNF from neurons or astrocytes in a neuron–astrocyte coculture system after OGD/R. The results showed that neurons and astrocytes can mutually promote the release of BDNF in the coculture system. Astrocytes become an important source of BDNF because astrocytes were in an activated state, while damaged neurons reduced the release of BDNF. As shown in Figure 11, BDNF released by reactive astrocytes in the coculture system were an important cause of anti-neuronal apoptosis and damage after OGD/R. BCL treatment can maintain the activated state of astrocytes and increase BDNF levels in the NA coculture system. BDNF expression is regulated by CREB. Deregulation of CREB indicates that a deficiency in neurotrophic factor synthesis and signaling underlies IS. BCL treatment can increase the expression of CREB, which further promotes the release of BDNF. BCL-promoted release of endogenous BDNF has important clinical significance. Previous studies focused on developing exogenous small-molecule mimetics of BDNF. However, because of the short serum half-life, poor bioavailability, extremely low BBB penetration, and limited diffusion within CNS tissue, exogenous BDNF has been proven to have certain problems clinically. Therefore, it is reasonable to enhance further the expression of BDNF from neurons and reactive astrocytes by pharmacological treatments.

Additionally, BCL treatment increased downstream activation of its cognate TrkB receptors, leading to the hypothesis that neuroprotection provided by BCL to the NA coculture system damaged by OGD/R is mediated by BDNF. It has been well established that BCL effectively exerts neuroprotective activity in *in vitro* and *in vivo* models, including antioxidant, antiapoptotic, anti-inflammatory, and anti-excitotoxicity effects; protection of the mitochondria; promotion of neuronal protective factor expression; and adult neurogenesis effects (Cao et al., 2011; Zhu et al., 2012; Fafeng et al., 2013; Liang et al., 2017; Kandhasamy et al., 2018). To test this hypothesis, we asked whether downstream activation of TrkB receptors by BDNF was required for neuroprotection by BCL. First, we took a pharmacological approach using an antagonist of high-affinity neurotrophin TrkB receptors, ANA12. The present study found that BCL could attenuate OGD/R-related pathological mechanisms, including free radicals, inflammation, and apoptosis, whose effects were decreased by ANA12. However, the protective effect of baicalin on the coculture system was not completely mediated by BDNF/TrkB, and it can also directly resist oxidative stress, inflammation, and apoptosis. Moreover, activation of three downstream targets of BDNF/TrkB signaling (p-ERK, p-CREB, and p-Akt) was increased in the NA coculture system by BCL treatment. ANA12 also decreased the effects of BCL on the expressions of p-ERK1/2, p-Akt, and p-CREB in the NA coculture system. The PI3K/Akt and MAPK/ERK1/2 cascades are pivotal signaling pathways that promote neuronal survival and plasticity to provide essential protective effects on neuronal activity after IS (Liu et al., 2020). Similarly, the activation of PI3K, Akt, MAPK, and ERK by BCL treatment was not completely blocked, and they can also be activated through other pathways. A large number of previous studies have proven that BCL plays a role in resisting oxidative stress, inflammation, and apoptosis through a large number of pathways, including TLR4/NF-κB, JAK2/STAT3, and PPARγ (Liang et al., 2017). Therefore, BDNF-TrkB/PI3K/AKT and BDNF-TrkB/MAPK/ERK were only part of the protective mechanism.

In summary, the present work identified astrocytes as an important source of BDNF after OGD/R. BCL upregulated the release of BDNF and the expression of proteins related to the BDNF-TrkB pathway in the neuron–astrocyte coculture system and that blockade of the BDNF-TrkB pathway reversed the protective effect of BCL against OGD/R-induced oxidative stress, inflammation, and apoptosis. These results suggest that the BDNF-TrkB pathway may be a newly contributory mechanism to the protective effects of BCL against OGD/R-induced injury paradigms. The present study could provide a potential clinical drug that can protect multiple mechanisms, protect multiple cells, and promote neuronal recovery through astrocytes.

## Data Availability

The original contributions presented in the study are included in the article; further inquiries can be directed to the corresponding authors.
