# Supplementary material for: Baicalin Attenuates Oxygen–Glucose Deprivation/Reoxygenation–Induced Injury by Modulating the BDNF-TrkB/PI3K/Akt and MAPK/Erk1/2 Signaling Axes in Neuron–Astrocyte Cocultures
Source: Front Pharmacol. 2021 Jun 21;12:599543. doi: 10.3389/fphar.2021.599543 (PMC8255628; doi:10.3389/fphar.2021.599543)
Supplement: Supplementary file 2 [file DataSheet1.PDF]

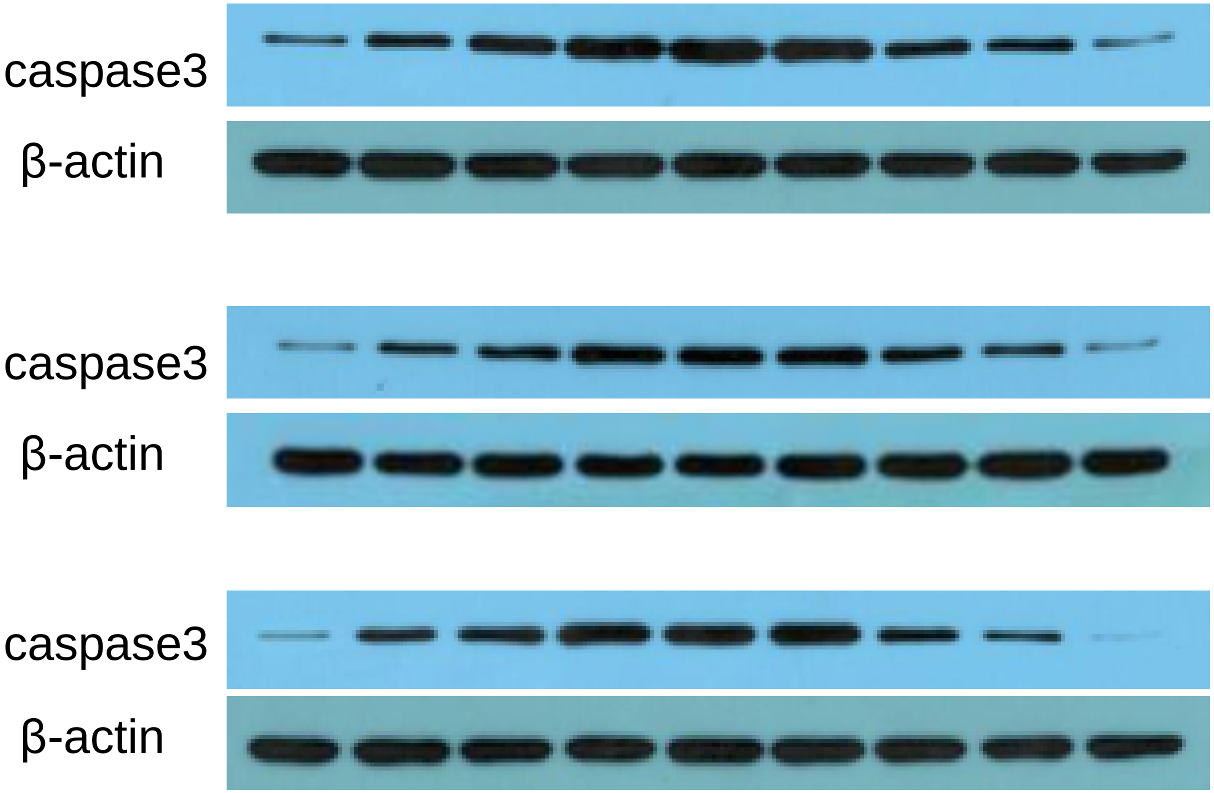
caspase3
β-actin
caspase3
β-actin
caspase3
β-actin

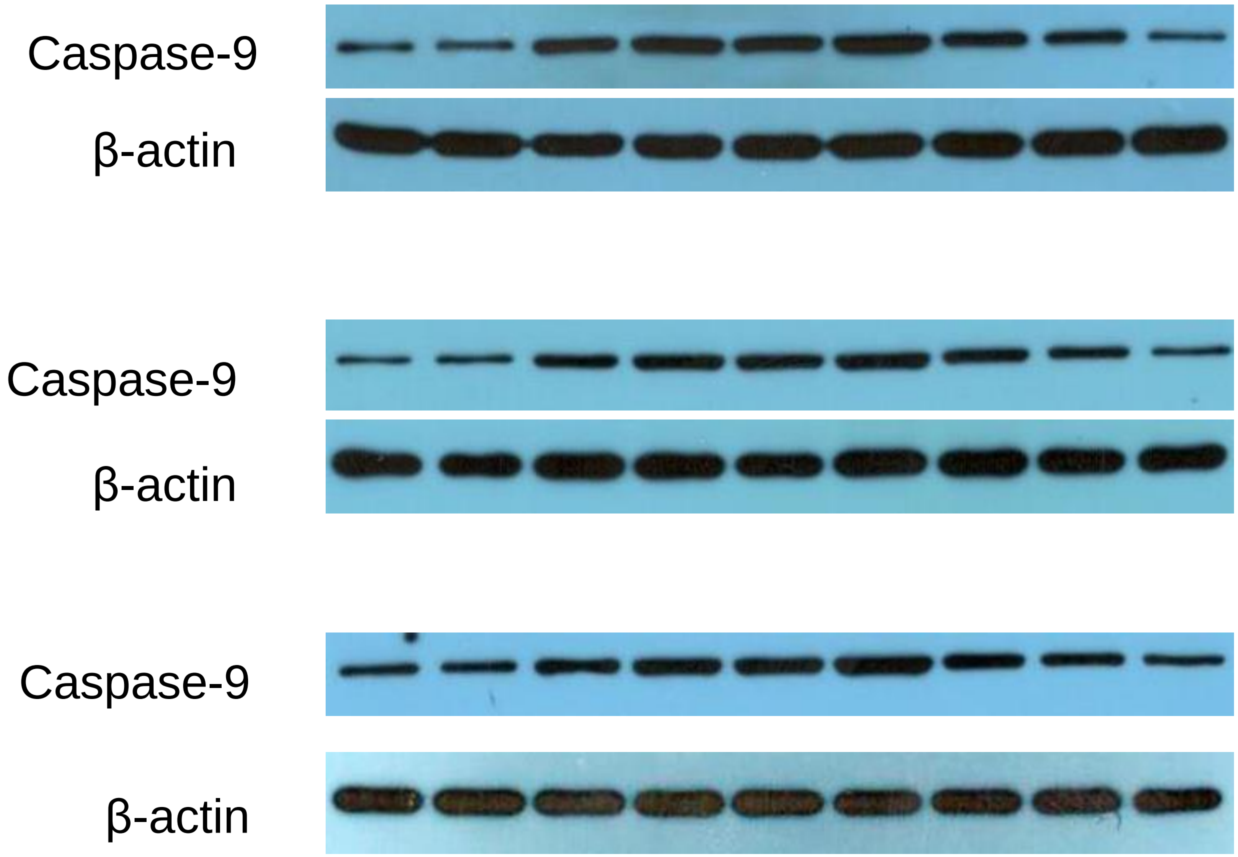

Caspase-9
β-actin
Caspase-9
β-actin
Caspase-9
β-actin

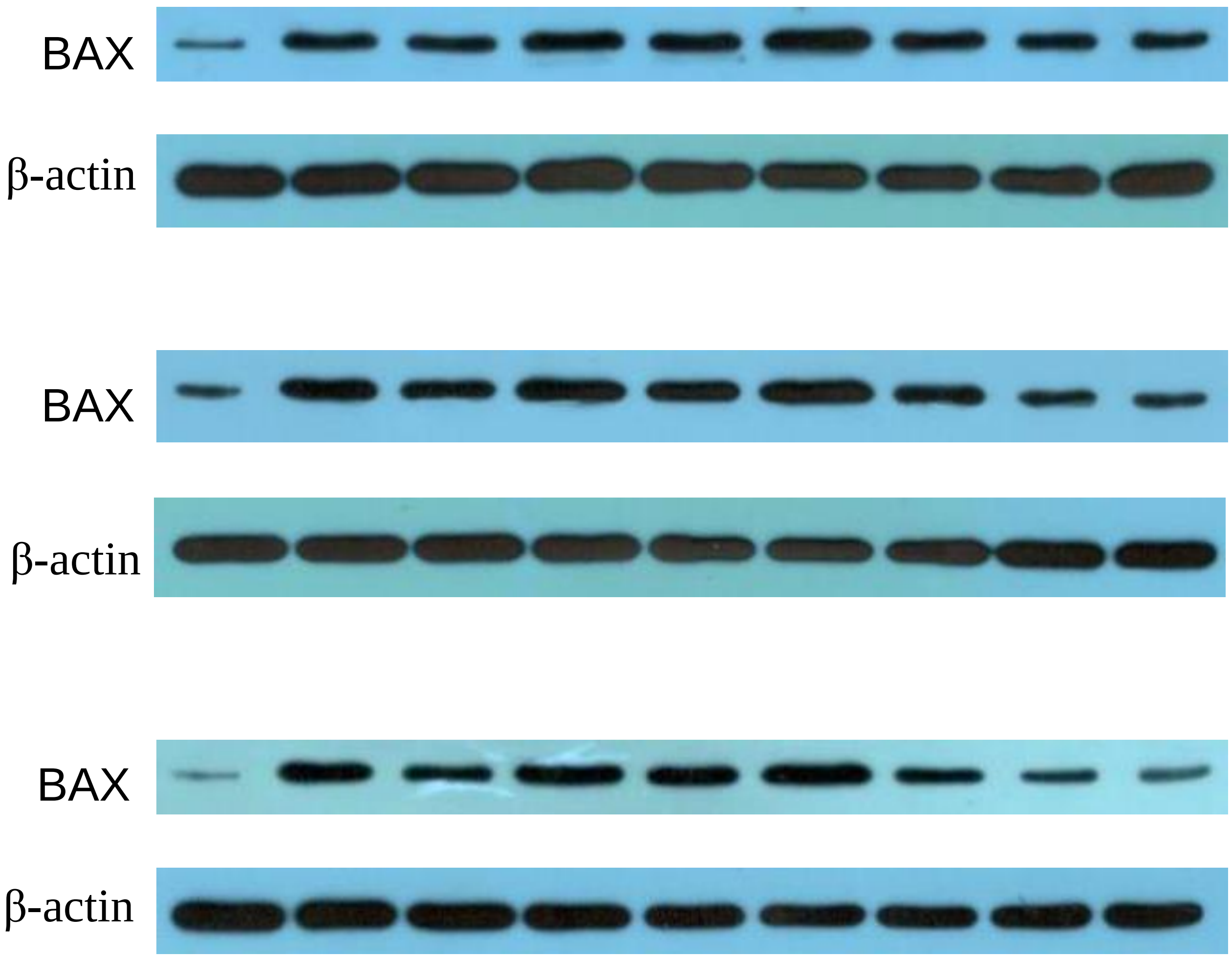
BAX
β-actin
BAX
β-actin
BAX
β-actin

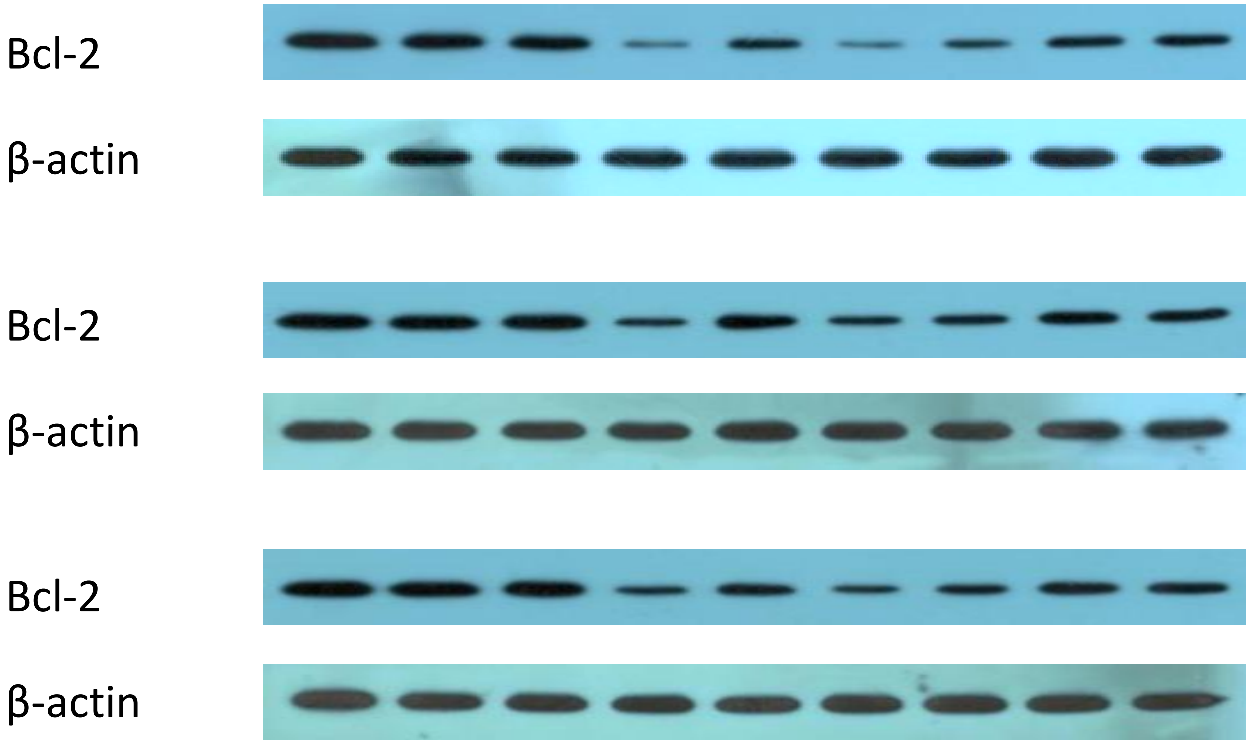
Bcl-2
β-actin
Bcl-2
β-actin
Bcl-2
β-actin

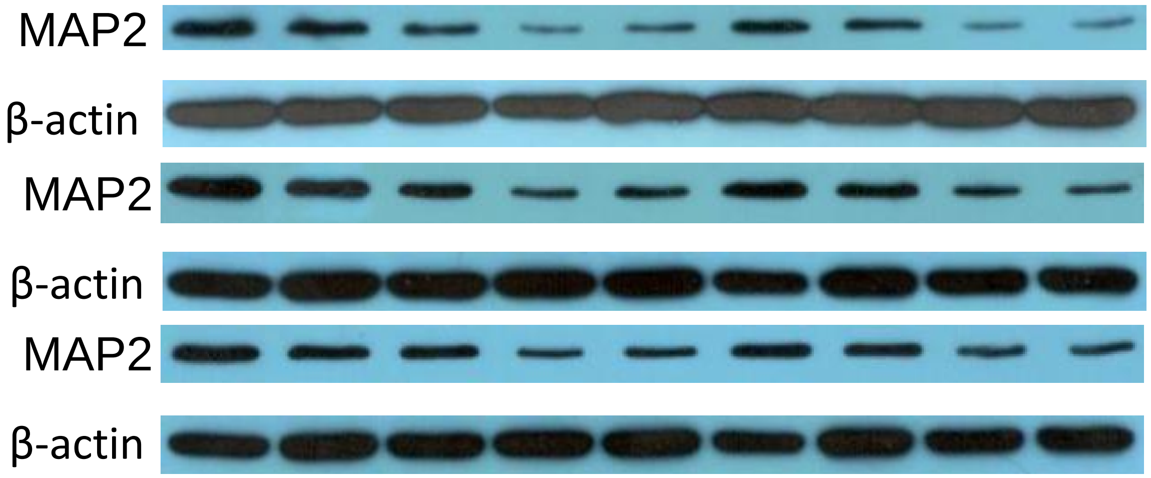
MAP2
β-actin
MAP2
β-actin
MAP2
β-actin

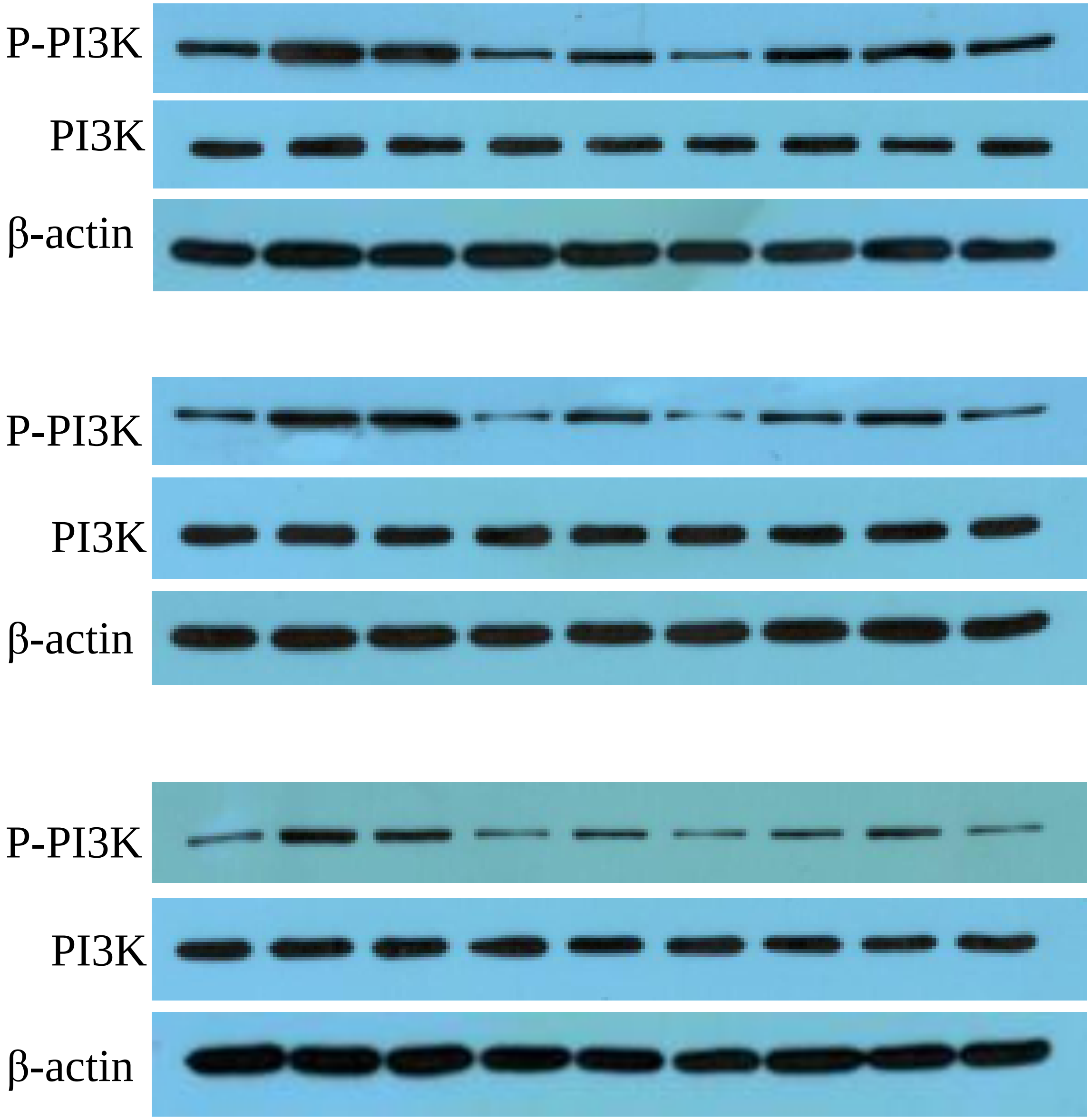
P-PI3K
PI3K
β-actin
P-PI3K
PI3K
β-actin
P-PI3K
PI3K
β-actin

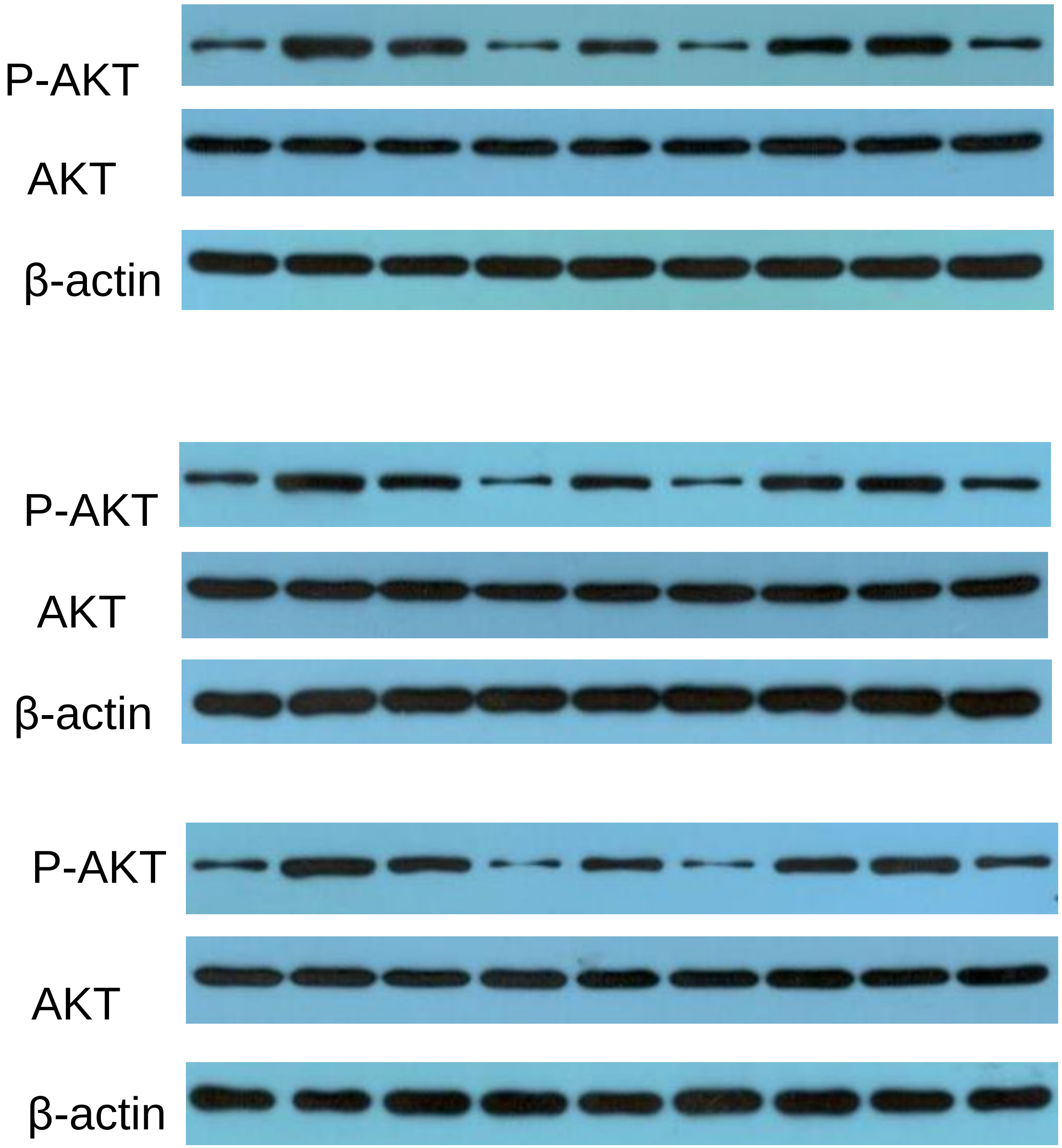
P-AKT
AKT
β-actin
P-AKT
AKT
β-actin
P-AKT
AKT
β-actin

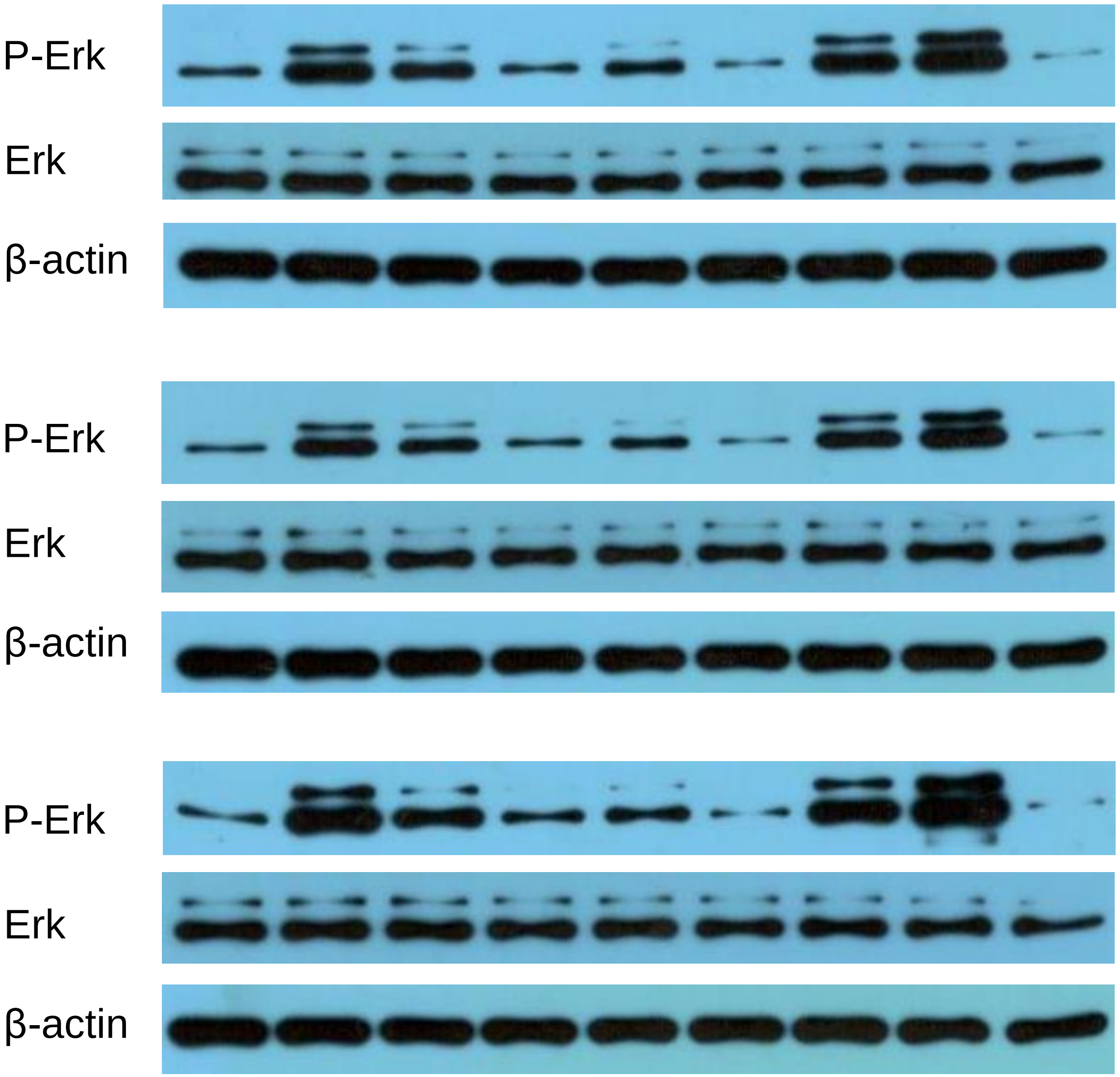
P-Erk
Erk
β-actin
P-Erk
Erk
β-actin
P-Erk
Erk
β-actin

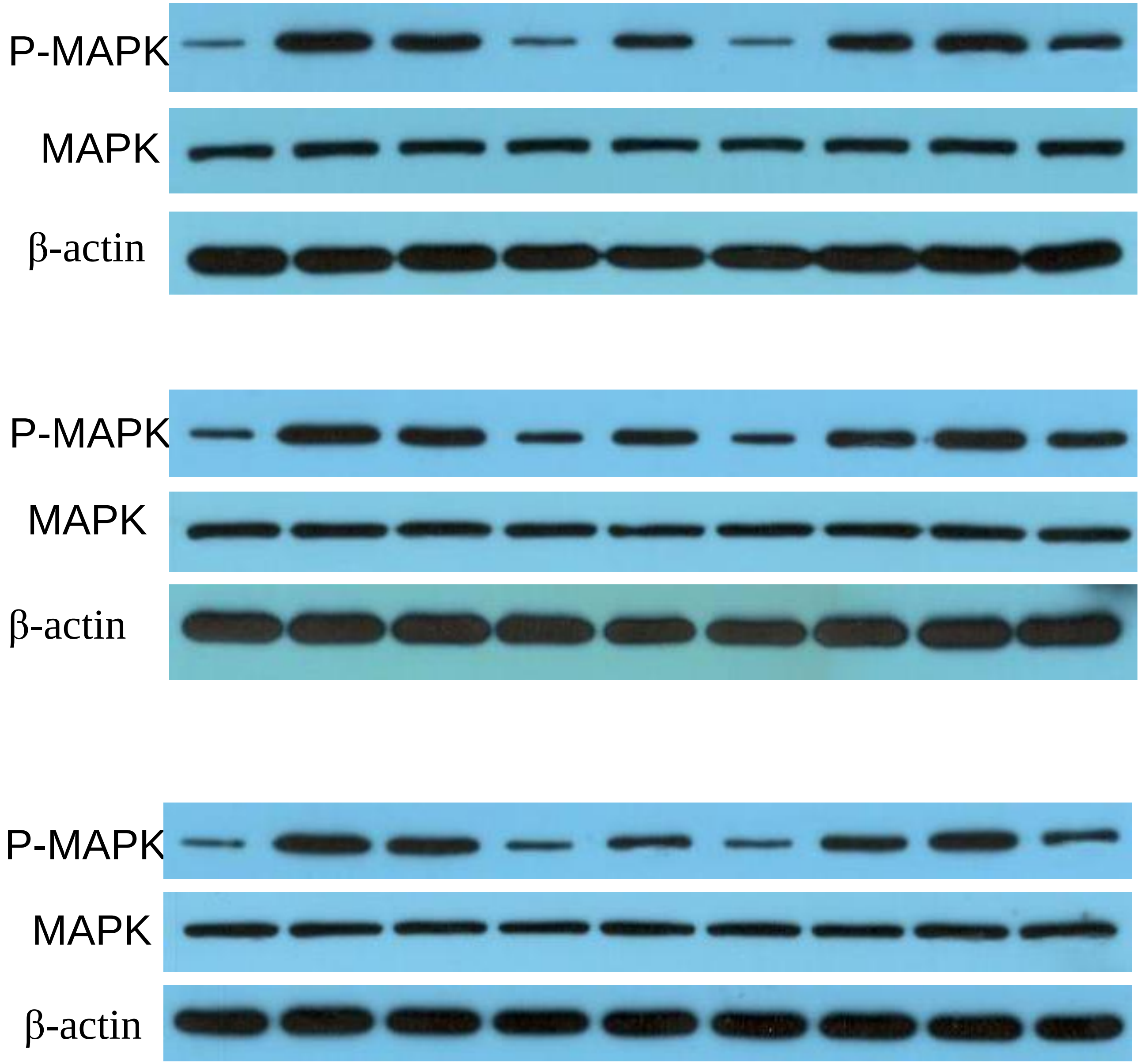
P-MAPK
MAPK
β-actin
P-MAPK
MAPK
β-actin
P-MAPK
MAPK
β-actin
